# Quantifying Motor Impairment in Movement Disorders

**DOI:** 10.3389/fnins.2018.00202

**Published:** 2018-04-11

**Authors:** James J. FitzGerald, Zhongjiao Lu, Prem Jareonsettasin, Chrystalina A. Antoniades

**Affiliations:** ^1^NeuroMetrology Lab, Nuffield Department of Clinical Neurosciences, University of Oxford, Oxford, United Kingdom; ^2^Nuffield Department of Surgical Sciences, University of Oxford, Oxford, United Kingdom; ^3^Department of Neurology, West China Hospital of Medicine, Sichuan University, Chengdu, China; ^4^Exeter College, University of Oxford, Oxford, United Kingdom

**Keywords:** quantification, neurosciences, eye trackers, accelerometers, technologies, rating scales, movement disorders

## Abstract

Until recently the assessment of many movement disorders has relied on clinical rating scales that despite careful design are inherently subjective and non-linear. This makes accurate and truly observer-independent quantification difficult and limits the use of sensitive parametric statistical methods. At last, devices capable of measuring neurological problems quantitatively are becoming readily available. Examples include the use of oculometers to measure eye movements and accelerometers to measure tremor. Many applications are being developed for use on smartphones. The benefits include not just more accurate disease quantification, but also consistency of data for longitudinal studies, accurate stratification of patients for entry into trials, and the possibility of automated data capture for remote follow-up. In this mini review, we will look at movement disorders with a particular focus on Parkinson's disease, describe some of the limitations of existing clinical evaluation tools, and illustrate the ways in which objective metrics have already been successful.

## Introduction

**Figure 1:**
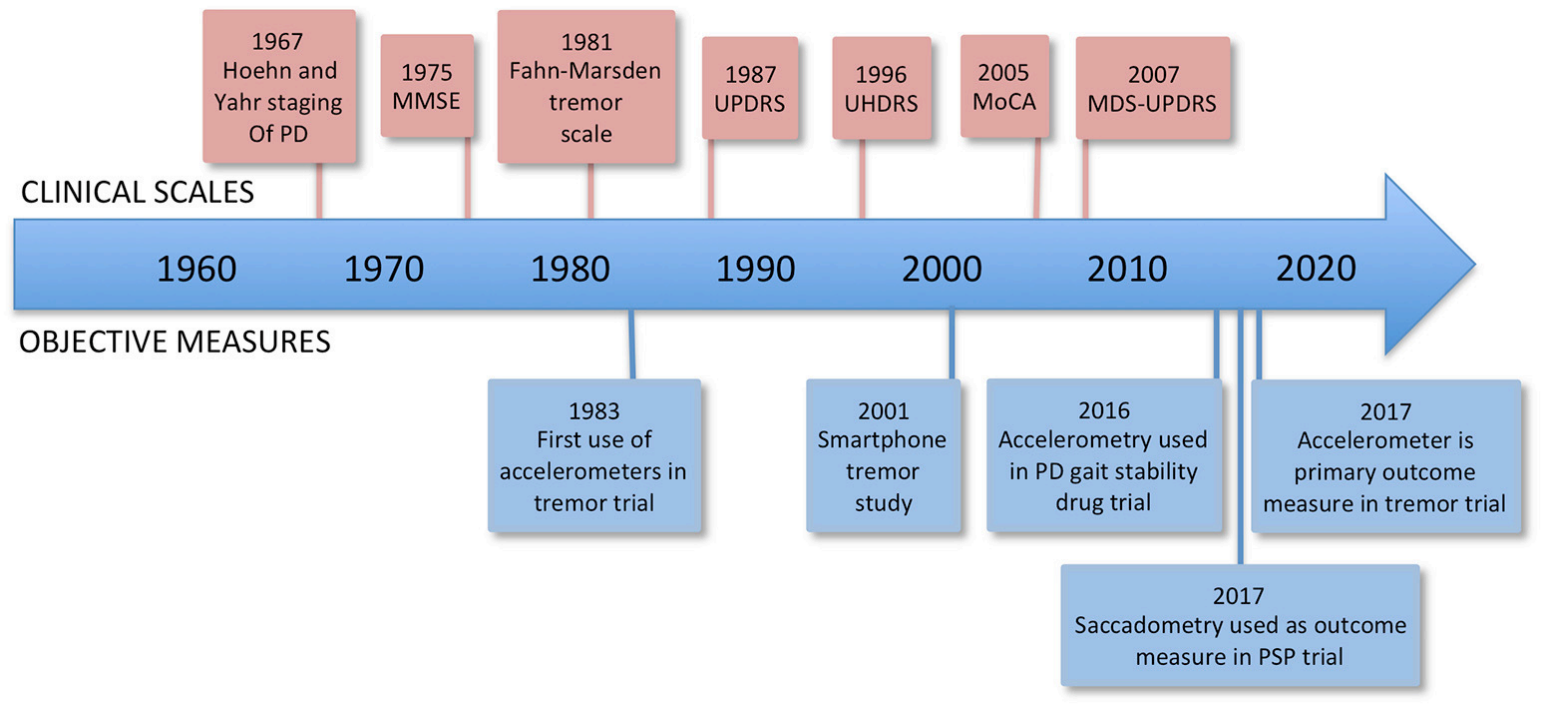
Timeline of rating scales and significant firsts in the use of measurement technology. MMSE, Mini-Mental State Examination; UPDRS, Unified Parkinson's Disease Rating Scale; UHDRS, Unified Huntington's Disease Rating Scale; MoCA, Montreal Cognitive Assessment; MDS-UPDRS, Movement Disorders Society Unified Parkinson's Disease Rating Scale.

One of the problems in trying to correctly diagnose and treat brain diseases, as well as conduct clinical trials of new treatments, is that at present we lack sensitive, objective, and quantitative measures of relevant aspects of brain function. The most widely accepted metric for most neurological conditions is a disease-specific clinical rating scale, for example the Movement Disorders Society Unified Parkinson's Disease Rating Scale (MDS-UPDRS) (UPDRS, 2003). Such scales usually involve an element of judgment by the rater and thus are not entirely objective, and they are not on an interval scale, complicating and limiting statistical analysis (see Figure 1).

There are several reasons why neurological conditions have often escaped precise objective enumeration. They can be intrinsically difficult things to measure, often being multifaceted conditions where measuring any one component accurately may not represent the patient's overall impression of “how bad” they are. Parkinson's disease for example may have ten or more symptoms present in varying combinations. Symptoms may vary over time, making snapshot measurements difficult to interpret. Importantly, many neurological conditions lack effective disease-modifying treatments. When the only treatments available are symptomatic, all that matters in clinical practice is whether the patient feels better taking them, a simple question requiring no measurement to answer.

This situation is particularly unsatisfactory when testing new potentially disease modifying treatments in clinical trials. It is critical that the measures used to evaluate them are as sensitive, objective, and free of noise as possible, both in order to select and stratify trial entrants, and to accurately gauge the results. Pharmaceutical trials consume vast amounts of time and money, and only one in ten drugs entering trials makes it all the way through to regulatory approval (Hay et al., 2014). Thus while it is essential to accurately identify effective drugs, it is just as important to be able to quickly kill off research into agents destined to fail, so that time and money can be transferred to alternative targets. The ability to rapidly and reliably appraise candidate agents in futility trials is a key performance gap that needs to be addressed. Motor UPDRS change at 1 year in unmedicated PD patients appears to be an appropriate measure in some cases. However, it is confounded completely by the presence of symptomatic dopaminergic treatments (Elm et al., 2005). The ideal measure would operate at a shorter timescale and be resistant to these confounding effects.

## Rating scales and their problems

### Non-linearity

Clinical rating scales are typically integer based and are on an ordinal rather than interval scale. If condition X is measured on a scale of 0–100, one cannot assume that the difference in severity between scores of 30 and 40 is the same as the difference between scores of 20 and 30. Non-parametric methods are therefore recommended when analysing rating scale data (UPDRS, 2003), which may be less statistically powerful than their parametric counterparts.

### Multidimensionality

Rating scales are commonly multi-item, i.e., made up of several component scores that are added. In a well-designed scale, although there are multiple questions they are all ultimately assessing the same thing (it is “unidimensional”), and the questions are constructed so that they each approach the same issue in a somewhat different way. This minimizes the effect of variability in grading or interpretation of individual scale items. Not all scales have been designed with such principles in mind from the outset, and dependence on rating scales that have not been meticulously developed can undermine the interpretability of study results. Hobart (2003) cites as an example of this the DATATOP trial (DATATOP, 1989), a major clinical study of the effects of selegiline as a potential neuroprotective agent in Parkinson's disease, which may have been compromised by its reliance on the UPDRS: “*Unfortunately, the unified Parkinson's disease rating scale, the primary outcome measure in the DATATOP study, confounds symptoms with disabilities…the UPDRS was developed without established techniques of rating scale construction.”* Even just the motor subscore (part III) of the UPDRS is not unidimensional; in fact, analysis suggests that it is measuring four different things (Stochl et al., 2008).

### Ceiling and floor effects

Rating scales may suffer from insensitivity at their upper or lower extremes. A good illustration of this is the Montreal Cognitive Assessment (MoCA) when used in early PD patients. The MoCA is a well validated tool and analysis shows that in this setting the MoCA is a unidimensional measure of global cognitive impairment (Kletzel et al., 2017). However, there is ceiling effect: most patients get scores at or near to the top end of the scale. In this study (Kletzel et al., 2017), 80% scored in the unimpaired range, however it is clear from studies using more difficult tasks, for example the trail making task and anti-saccadic task, that cognitive performance in many newly diagnosed and unmedicated patients falls well below that of controls (Antoniades et al., 2015a). Put simply, for this group the questions in the MoCA are not hard enough.

### Inter-observer variability

No matter how well designed a clinical rating scale is, there will inevitably be some subjectivity. The UPDRS can exhibit considerable inter-rater disagreement (Post et al., 2005). Becoming fully familiar with complex scales may necessitate specialist training and considerable subsequent practice, but this in turn can also limit their accessibility.

## Improving rating scale data

Methods have been developed to transform rating scale data onto an interval scale. Rasch analysis (RA) is one such method and this has been applied to some scales used in Parkinsonism. It is important to note however that no transformation can compensate for problems with scale design, such as multidimensionality. For example, application of RA to the Parkinson's disease quality of life instrument PDQ-39 shows that it is not unidimensional (Hagell and Nilsson, 2009), whereas a similar evaluation of the carers' quality of life in parkinsonism scale (Pillas et al., 2016a) shows that it is unidimensional, and thus only the second of these two measures is amenable to transformation to an interval scale (Pillas et al., 2016b).

The UPDRS is also not unidimensional (UPDRS, 2003), however a recent study applying RA to just the 11 tremor related elements in the UPDRS showed that they do form a unidimensional scale, and moreover because of redundancy they can be reduced to just seven items without loss of information (Forjaz et al., 2015).

In situations where measurement of concrete physical variables is never likely to be possible, for example where we are trying to measure quality of life, this type of analysis coupled to a well-designed scale is likely to be the best way forward. Where there are real physical variables to measure however, technology is eventually likely to take over. We now consider two rapidly developing examples of the use of quantitative measuring technology in movement disorders research and practice.

## Technologies

### Accelerometers

Accelerometers are the key component in wearable actigraphic devices. They can detect magnitude and direction of acceleration, vibration, and shock, and orientation with respect to gravity. Many newer sensors additionally integrate polyaxial gyroscopes to measure rotational movements. Devices combining linear accelerometers and gyroscopes are referred to as inertial measurement units (IMUs).

Perhaps the most obvious application of accelerometers in neurology is measuring tremor. In fact accelerometers have been used since the 1980s in clinical trials assessing the effects of drugs on essential tremor (Baruzzi et al., 1983; Koller and Vetere-Overfield, 1989). Tremor is characterized by its frequency spectrum and its amplitude, and an accelerometer can precisely enumerate both. The use of smartphones to analyse tremor syndromes was demonstrated in 2001 (Joundi et al., 2011), using an application initially intended as a seismometer. This provides the clinician at zero cost (provided they have a telephone) with an instant alternative to electromyographic tremor studies. Spectral analysis of accelerometer (Hossen et al., 2013) and gyroscopic (Bhidayasiri et al., 2014) measurements can discriminate between PD tremor and Essential Tremor (ET) or between idiopathic and drug-induced parkinsonism (Jang et al., 2013). Accelerometer studies have even attempted to subdivide these conditions, for example separating patients with essential tremor into those that are position-dependent vs. position-independent (Golan et al., 2004), or differentiating tremor frequency characteristics of PD patients under resting vs. stressed states (Lee et al., 2016).

Accelerometers or IMUs have been used to analyse gait in conditions including Parkinson's disease (Dijkstra et al., 2010; Bryant et al., 2011; Fazio et al., 2013; Hatanaka et al., 2016), Huntington's disease (HD) (Andrzejewski et al., 2016), cerebellar ataxia (Shirai et al., 2015), dementia (Ijmker and Lamoth, 2012), and stroke (Dobkin et al., 2011). The gait of patients with progressive supranuclear palsy (PSP) is differentiable from that of Parkinson's disease on the basis that while both show similar hypokinetic gait, PSP patients show reduced vertical displacement (Hatanaka et al., 2016). It is also possible to differentiate parkinsonian vs. ataxic gait (Fazio et al., 2013), and parkinsonian vs. dementia gait (Yoneyama et al., 2016). Accelerometry has recently been used in a randomized trial assessing the effects of rivastigmine on gait stability in PD (Henderson et al., 2016).

The datasets yielded by inertial measurement systems can be enormous. A body worn array of IMUs can easily stream tens of megabytes of data per minute from a walking subject. The number of gait parameters that can be extracted is large, and there are additional variables to consider, related to the environment and instructions given to the subject (Vienne et al., 2017), for example the nature of the surface they are walking on, the pace they are asked to walk at (Bryant et al., 2011), and the overall environment (laboratory or home; Dijkstra et al., 2010). Simple parameters such as gait speed or cadence are easy to analyse, but making use of the full richness of the dataset is likely to require advanced computational methods such as machine learning. Examples include applying a Bayesian classifier to bilateral ankle accelerometer data in order to recognize walking, exercise, and cycling activities during rehabilitation of stroke patients (Dobkin et al., 2011), and analysis of belt-worn accelerometer data using support vector machines to look for signs of freezing of gait (Rodriguez-Martin et al., 2017) and dyskinesia (Perez-Lopez et al., 2016) in PD.

A recent review in PD (Godinho et al., 2016) identified 73 different measuring technologies, 22 of them wearable. Some of these technologies are being used to replace clinical assessment of components of rating scales with electronic measurements of the same things, eliminating inter-observer variability. For example, the finger-tapping element of the MDS-UPDRS can be predicted by accelerometer data (Stamatakis et al., 2013). Studies using accelerometers have also suggested measuring things that are not presently included in any rating scale, such as mediolateral sway (Mancini et al., 2012), which may be a marker of PD progression. Accelerometry may also be able to recognize disease early in its course, when prodromal symptoms are below the floor of the usual rating scales, or even before the condition is manifest. Lumbar accelerometers detect increased variability of trunk acceleration and smoothness of sway in subjects known to be at risk of developing PD (Maetzler et al., 2012).

In recent years, studies have started using portable kinematic systems to quantify PD motor deficits alongside to clinical rating scales. Such kinematic systems have shown greater test-retest reliability and sensitivity than the clinical rating scales particularly for parameters of bradykinesia, hyperkinesia, and dysrhythmia (Heldman et al., 2014). Quantitative kinematic variables are highly correlated with a bradykinesia score (Heldman et al., 2011; Matias et al., 2017).

Accelerometers are beginning to be used in assistive technologies as a component part of treatment itself. Accelerometer data can be used to provide feedback in visual/auditory cueing devices for gait-impaired PD patients, so that the sensory cues are coordinated with the patient's gait cycle (Espay et al., 2010), improving performance when compared to cues that are not patient-driven in this way.

### Eye movement recording

Extraocular muscle control is complex and involves multiple brain areas, including the cerebellum, the brainstem, cerebral cortex, and the basal ganglia (Kennard and Leigh, 2008). Because of this, oculomotor function can be affected by many different pathologies (Antoniades and Kennard, 2014; MacAskill and Anderson, 2016). One would expect that as a disease progresses, so should the eye movement abnormalities that it produces, and also that each disease might produce a different signature pattern of eye movement changes. This has led to the idea that eye movements could be a biomarker for both diagnostic purposes and for monitoring disease progression or response to treatment.

Early oculography relied on objects attached to the eye. In 1908, Huey described a device that featured an eye cup worn like a contact lens, attached to a lever which made marks on a smoked drum (Huey, 1908). Another design involved grinding plane mirror elements on the lenses and recording light reflected from them on photosensitive paper (Ditchburn and Ginsborg, 1953). In 1963, Robinson introduced the scleral search coil (Robinson, 1963), a contact lens containing a built in wire coil that picks up an ambient electromagnetic field produced by larger coils placed around the subject. All these methods are invasive and uncomfortable. Most have disappeared, except for scleral search coils which are still used in some neurophysiology research laboratories because of their exquisite temporal and spatial resolution, and their ability to accurately measure eyeball rotation around all three axes.

Electrooculography (EOG) (Mowrer et al., 1936) measures changes in the orientation of the corneo-retinal electric dipole (an electric field that is produced by physiological activity within the retinal pigment epithelium), using skin surface electrodes positioned around the orbits. It gives excellent time resolution for fast movements (saccades) but is less good for determining absolute eye position. It can be measured with the eyes shut and has thus been used in studying REM sleep behavior disorder (RBD) (Kempfner et al., 2011), a condition that can presage the development of PD.

Most modern eye tracking uses reflected light technology or video oculography (VOG). Reflected light devices typically analyze the reflection of (often infra-red) light from the corneal reflex or the limbus (Torok et al., 1951), or Purkinje images, reflections from structures at various depths in the pupil (Crane and Steele, 1985). VOG employs computerized analysis of video recordings to follow pupil position. Several good detailed reviews of historical and current eye tracking technology have been published elsewhere (Wade and Tatler, 2005; Eggert, 2007).

Saccades, the rapid eye movements that shift the fovea to objects of interest, have been the most intensively studied type of eye movement in biomarker research. The simplest type, the prosaccade, exhibits abnormalities in a wide range of conditions. Prosaccadic latency (PSL, the time taken to initiate a saccade to a novel stimulus) is prolonged in several disorders of the basal ganglia including PD (Armstrong et al., 2002; MacAskill et al., 2002; Chan et al., 2005; Terao et al., 2013), PSP (Vidailhet et al., 1999; Antoniades et al., 2007b), and HD (Lasker et al., 1987; Blekher et al., 2004; Antoniades et al., 2007a; Peltsch et al., 2008; Biglan et al., 2009; Rupp et al., 2010; Wiecki et al., 2016). The prolongation progresses over time. The variability of latency within each individual is extremely informative and can be described by parameters of a subject's reaction time distribution plot (Carpenter, 1994). The pattern of changes in these parameters can be used to differentiate between conditions such as PD, PSP, and atypical parkinsonian syndromes (Antoniades et al., 2007b).

In PD, the effects of treatment can be observed in changes in PSL, but in a perhaps surprising way: although levodopa can improve the symptoms of PD, it lengthens PSL even more (Hood et al., 2007). Interestingly, deep brain stimulation of the subthalamic nucleus (STN) or globus pallidus pars interna (GPi) also improves symptoms yet does the opposite to PSL, shortening it (Temel et al., 2008; Antoniades et al., 2012). These observations may hold valuable insights into disease pathophysiology, treatment mechanism, or both.

More complex paradigms can be used to tease out and measure deficits in higher functions. The antisaccade task (Hallett, 1978; Rupp et al., 2011; Cordones et al., 2013; Antoniades et al., 2015b) for example requires both response inhibition and volitional saccade generation, both frontal lobe functions. This task has been used to measure cognitive decline in very early stage PD, when standard cognitive scales were insufficiently sensitive to pick up any impairment (Antoniades et al., 2015a). In HD, where gene carriers can be identified with a blood test so that we know who is likely to get the disease, a computational study has found abnormalities in antisaccadic behavior even at the premanifest stage (Wiecki et al., 2016). In one of our recent studies of more advanced PD patients (Antoniades et al., 2015b), we have shown that while DBS to GPi and STN both reduced latency to prosaccades, only DBS to GPi improved antisaccadic performance. The discovery was the first direct evidence that DBS could improve higher control of motor functions in Parkinson's disease.

Eye movements are nowadays easily measured using computerized high-speed eye trackers. These have high temporal resolution and spatial precision and have sampling frequencies of 1,000 HZ. Furthermore, technological advances have introduced head-mounted tracking which not only increases the portability of the eye trackers but also the feasibility in a clinical environment. Many of these portable eye trackers are also easy to programme and therefore parameters such as latency and stimulus location can be altered according to the clinical application.

## Cautions

Devices such as accelerometers can measure physiological variables with great precision. It does not necessarily follow that they give an accurate representation of the patient's overall condition. Many neurological symptoms vary profoundly by time of day, medication timing, or seemingly randomly, and isolated “snapshot” measurements may be of limited value. Tremor amplitude is a good example. In a study of essential tremor, using serial accelerometric measurements under standardized conditions, 17 of 22 patients showed a coefficient of variability in their tremor amplitude of at least 25% (Cleeves and Findley, 1987). Furthermore, a comparison of postural accelerometry with rating scale evaluations such as examination of writing or drawing spirals found that the latter methods produced better correlation with self-reported disability than accelerometer measurements (Bain et al., 1993). One solution to these problems may be the use of extended ambulatory measurements to capture a fuller picture of the tremor and its variability.

Isolated measurements are not without value however. Although tremor *amplitude* is highly variable, tremor *frequency* is not, and indeed in most cases it is constant to within a range of <1 Hz (Cleeves and Findley, 1987). Frequency information provides clues about the cause of the tremor, and diagnostically useful data may therefore be obtained even from single measurements.

In order for new measuring technologies to gain acceptance by medical professionals and researchers, they must be validated in clinical studies. To be useful in clinical trials they also need to be accepted by regulatory authorities. When there is no entirely reliable “gold standard” measure to compare it to, validating a new test is not straightforward and it generally requires several supportive studies before acceptance becomes widespread. There has been a proliferation of measuring devices and algorithms (Godinho et al., 2016) with widely varying degrees of validation. Selection of measurement techniques for a new study is a much more complex task than it used to be, yet is of vital importance to the generalizability of the results. Researchers must therefore choose carefully.

## Future directions

Device-based quantitative measures are gradually taking on a greater role in movement disorders research and treatment. Accelerometry is the primary outcome measure, in one ongoing RCT of a novel drug in ET, with a tremor rating scale as a secondary measure (clinicaltrials.gov/ct2/show/NCT02978781). Eye movement measurement is at an earlier stage of this process, and to our knowledge there are as yet no trials using eye movement measurements as a primary outcome, but saccadometry is a secondary outcome measure in a current drug trial in PSP (clinicaltrials.gov/ct2/show/NCT01056965).

Efforts are currently underway to assemble very large sets of sensor data in PD, with the intention that these will in future be available on an open source basis as a resource to researchers. Such large datasets will require “big data” analytic techniques to mine them. One example (Cohen et al., 2016) uses a consumer wrist-worn triaxial accelerometer that sends data to the cloud via an app on the user's smartphone; a large number of patients can then submit continuous data. Ready availability of big datasets will accelerate the pace of research, although it is important to remember that the information that can be extracted from the data depends critically on how it was captured. A careful balance must be struck: simpler sensor systems will maximize participation, but lead to a less rich dataset for others to mine later.

Finally, once we have sensor-based metrics that are widely accepted, taken using either consumer electronic devices or clinical equipment inexpensive enough to give to patients for home monitoring, the need for a clinician to be present to rate the patient's condition disappears. These developments will greatly increase remote telemedicine, and studies are already underway looking at remote monitoring of PD symptoms (Heldman et al., 2016).

## Author contributions

CAA and JF: designed the draft for this paper; ZL: contributed with the preparation of the figure and table and a revised draft; PJ: contributed on a revised draft. All authors contributed to and had approved the final manuscript.

### Conflict of interest statement

The authors declare that the research was conducted in the absence of any commercial or financial relationships that could be construed as a potential conflict of interest.
